# A Practical Treatise on Diseases of the Eye

**Published:** 1844-10-01

**Authors:** 


### A PRACTICAL TREATISE ON THE DISEASES OF THE EYE. By *William Jeaffreson*, late Surgeon to the Bombay Eye Infirmary, in the Hon. East India Company's Service, &c. &c. London. Renshaw, 8vo. pp. 307.

THE author tells us that "the present work has been undertaken chiefly with the motive of inculcating the necessity for regarding ophthalmic practice as but a branch of the whole science of practical medicine, and in order to modify those more violent notions respecting the treatment of diseases of the eye, which are as yet even but too prevalent." We had really thought that no such necessity existed; and that no one in the present day even dreamed of practising as an "oculist" in the acceptation of the term as it was understood fifty years ago. It is true that certain parties are prone to adopt exclusive and violent extremes in practice—at least if we may believe their writings, although our own experience has often shown us that many are more prejudiced on paper than when adapting their opinions to individual cases—yet we think that we shall prove in a subsequent page that our author would conduct any, who were blind enough to trust him, from one grave error into another as dangerous, if not more fatal to vision. Have the Travers', Lawrence's, Middlemore's and Tyrrell's of this country written in vain? that it is left to Mr. Jeaffreson to write a work chiefly to inculcate "the necessity for regarding ophthalmic practice as but a branch of the whole science of practical medicine." We had thought, indeed, that the profession were now fully persuaded that—in the words of Dr. Hocken*—"No one can possess scientific views of ophthalmic disease unless its study be based on comprehensive views of disease generally—hence all was dark and erroneous as long as this department of medicine was monopolized by the charlatan, and it has only within the last few years acquired its present clearness and accuracy. In the eye we may behold a miniature representation of all diseases; for here, says Dr. Latham (Med. Gaz. Vol. iii.,

---

* A Complete Condensed Practical Treatise on Ophthalmic Medicine, Part 1, p. 1.

p. 216) "Nature has displayed, as in a glass, all the little intimate details of her own wonder-working powers; her modes of disorganizing, and her modes of repairing; and the aids which she receives, and the impediments she sustains, from the right and wrong application of medical agents."

Our author goes on to remark that, "in the multitude of other occupations, and the studies which the regulations of the present day have imposed upon the general practitioner, it is hardly possible for him to have devoted much time and attention to the consideration of this particular branch of medicine during his pupilage, and yet, in after-life, the responsibility of many serious forms of disease of the eye will probably fall to his lot. It has been the author's endeavour so to simplify his subject as to render assistance to this highly valuable and intelligent body of practitioners."

Taking it for granted that there exists a large class amongst the medical profession who are desirous of acquiring a clear correct knowledge of ophthalmic disease and the best modes of treatment, without having either the time or the inclination to wade through the voluminous publications of British and Foreign authors on the subject; it becomes a question what kind of work would best supply the wants of this particular class—viz. students and medical men actively engaged in the practice of their profession. It is of no use to tell a person thoroughly ignorant of the subject that "when the iris is the chief seat of inflammation or much implicated in the process, another important adjunct in the treatment deserves attention," unless you give him some clear directions by which he may distinguish iritis from conjunctivitis. Of what benefit then is our author's work likely to be to those who are practically unacquainted with the subject—for whose convenience the book is professedly written—when they turn to its pages and find that not the slightest information is given of the symptoms or diagnostic indications, beyond some general remarks, in such important diseases as those of the iris, cornea, sclerotica, and retina. Passing by these matters, which we presume he considers unnecessary, he proceeds at once to the consideration of treatment. "In acute inflammations then of the iris, cornea, sclerotica and retina (if indeed such ever *occurs* in an isolated form), the first object of the practitioner is to check or subdue the violence of the inflammatory action."

We have received for review within a recent period two Works on the Diseases of the Eye, written to supply the wants of those whose time is much occupied. One of these—Mr. Walker's Oculist's Vade Mecum—we noticed in our April Number; the other is by Dr. Hocken, and is entitled "A Complete Condensed, Practical Treatise on Ophthalmic Medicine," to be completed in three Parts—the first Part having only yet appeared. Another work, on a similar plan, has been advertised as in course of preparation by Mr. W. Jones. These works differ—and we have little doubt that Mr. Jones's will differ from our author's, inasmuch as they give a clear, condensed, practical account of the diseases of the different tissues of the eye,—so divided as to facilitate the acquisition of knowledge, previous to any remarks on the value of remedies and the direction of treatment: and hence are of much greater use to those who are desirous of acquiring information which will prove valuable in the hour of need.

The work which is really calculated to supply the wants of the student and actively-engaged practitioner, should be in truth a just medium between the voluminous works already in circulation, and those small, incomplete volumes, which are nearly, if not altogether, useless—and should contain a careful digest of the opinions and practice of foreign as well as British authors. Dr. Hocken's Work is written on this plan—his object being to give " a complete digest of all that is known of the Diseases of the Eye, both from his own experience and from all sources, British and Foreign." " He has condensed his chapters as far as was compatible with a *complete practical* view of the subjects treated of," and, " at the same time, has endeavoured to introduce every useful, practical and interesting fact." " As the main object has been to render the volume a faithful guide in treatment, the author has introduced a chapter on the best modes of examining the eye in various diseases, of arriving at a just diagnosis, and of directing treatment on the most successful principles." We fully believe that to examine an eye properly, to diagnose its diseases readily, and to have some clear general principles by which treatment shall be guided, is more than half the battle; and that common sense will generally be sufficient to guide the intelligent practitioner in the adaptation of remedial measures to particular cases, who is otherwise ignorant of ophthalmic practice ; and, on the contrary, that no one is competent to treat the diseases of the eye who is unable to form a proper opinion of their nature and of the tissues involved.

Our author's " Contents" consist of eight chapters, whereof the 1st is introductory; the 2nd treats of " Acute Non-specific Inflammation." 3rd. " Chronic and Specific Inflammation." 4th. " Structural Changes the result of Inflammation—Affections of the Humours, &c." 5th. " Affections of the various Appendages of the Eye." 6th. " On the Affections of the Nerves of Vision." 7th. " Cataract." 8th. " Malignant Diseases—Mechanical Injuries, &c.—Means of Preserving the Eyesight." Of this mode of arrangement we may remark that, had we to compose a work on the diseases of the eye, we should certainly take a lesson from it—a lesson, however, which would lead us to avoid and not to copy our author's kaleidoscope plan.

Our author gives a table of 53359 cases of ophthalmic disease which came under his care during the ten years he was surgeon to the Bombay Eye Infirmary—viz. from 1824 to 1834. " This table, constructed simply with the view of affording to Government a rough record of the total amount and leading characters of the cases which came under treatment, must not be considered in the same light, as if it had been drawn up for scientific purposes, in which respect it is certainly defective in many points of view." p. 12. " But of this number nearly seven thousand utterly blind persons were restored to perfect sight; namely, one thousand four hundred and thirty-two suffering from amaurosis, and five thousand five hundred and seventeen afflicted with cataract; the subjects of sixty-seven of which last were born blind." p. 16.

In another publication of the author's we find that the amaurotic cases were principally cured by internal remedies, and by the use of a native preparation, which, " strange to say, instead of being applied to the eye, is dropped into the ear !" At p. 47, the author tells us that " the natives of India are in the habit of using a liquid which they drop into the ear, in cases of ophthalmia, and which appears to owe its beneficial agency to a diametrically opposite mode of action to that of counter-irritation. No pain or inconvenience is experienced to the ear, and yet a soothing influence seems to be propagated to the eye. When first I heard of this practice I was disposed to consider it as altogether hypothetical: but I must confess that personal observation of its effects, in the hands of the native practitioners, as well as numerous subsequent trials made by myself, have induced me to believe this practice to be, in many cases, highly efficacious. When I explained to one of the native doctors, who employed this remedy, by means of a rough diagram, the nervous connexions between the eye and the ear, he could not conceal his satisfaction. I do not know the exact nature of this substance, but I suspect it to consist of narcotic and sedative ingredients. To the intimate relationship between the eye and ear, from the distribution of nervous filaments arising from a common trunk, is due, no doubt, the degree of sympathy so often observed to exist between these parts in various forms of disease, and to the same anatomical relationship is probably to be attributed the efficacy of this remedy, acting no doubt in the same inexplicable manner as many narcotic substances in ordinary use do, when applied externally for the relief of internal disorders. It may be remarked of this remedy, although deviating from the strict subject of our investigation, that it is no less highly esteemed by the native doctors in many forms of deafness; and I have myself had ample experience of its efficacy in some cases of this affection, having, during my residence in India, paid considerable attention to diseases of the ear as well as those of the eye."

From a careful perusal of our author's work, we are compelled to confess that it is beyond our power to find even one original opinion, of the least practical value to our readers; the whole being but an imperfect and confused account of matters which have been much more ably handled in almost every work on the eye, published during the last fifty years. Throughout the entire book the same lengthy but abstract reasoning on treatment is given—the treatment be it remembered of words and names —whilst, with one or two exceptions, symptoms are entirely omitted, it being, we presume, in the author's opinion, unnecessary for men actively engaged to know anything of ophthalmic disease, except in name—unnecessary to be able to distinguish one disease from another—and totally unnecessary to be so minute in diagnosis as to discriminate between the inflammations of the conjunctiva, cornea, iris, or retina. In the course of our painful journey through Mr. Jeaffreson's "Practical Treatise," whenever we have fallen on one portion which has pleased our wearied senses more than another, a striking similarity has caused us to turn to the work of some author with whose pages we were previously familiar, and left little doubt in our minds that, notwithstanding the immense experience of our author, he has not scrupled to avail himself of such assistance, although "the brevity and conciseness of the present work must plead an apology for that want of reference to other writers, which would be less excusable in a more lengthy treatise."

As our Journal proverbially deals as tenderly and favourably with the productions of authors as it can, consistently with a strict adherence to truth, we will conclude our notice of Mr. Jeaffreson's work by extracting a few of his remarks on treatment, winding up with the treatment of Cataract, which—although not novel—is, we think, the best part of the volume.

From our author's Preface to the concluding section, the tenor of his advice would, we think, have a tendency to lead the unwary from one grave error into another as injurious, if not more so, to vision. The errors we allude to are, on the one hand, unnecessarily active and violent methods of treatment (which our author especially reprobates); and, on the other, attending too much to the system at large, and too little to the suffering organ. Our author remarks:—

"Less has been trusted to nature, and diseases of the eye are even now combated with a severity of treatment which would not be applied to similar affections in other situations. True it is from the nature of the organ affected, slight changes alone, impairing the transparency of its structures, may be disastrous in a degree disproportioned to similar changes in other parts; but even in these structures, minute and delicate as they are, Nature is not deficient in her powers of repair; and the consideration of this subject should rather lead the public to seek early attention to diseases of the eye, than the profession to combat them at advanced stages, by means which are then at least incapable of restoring the structural alterations which have been already established. Then against this consideration must be set the still more important one—that the eye is not a vital organ—consequently but few of its diseases are attended with danger to life; the oculist therefore, should be careful in his attempts at repairing a valuable, but not a vital organ, to avoid as much as possible all serious injury to the whole machine, of which this is but a part."—*Preface.*

We fear that, whilst our author was soliloquising in an acute inflammation—say of the iris or retina—the organ would be irremediably lost for all useful purposes, and then the patient would find little consolation in being told that his system had escaped without injury, whilst vision was gone for ever. "Wisdom at one entrance quite shut out."

A few pages further on we find our author stating that, in acute inflammations of the iris, cornea, sclerotic and retina, "blood-letting may be required, but far less frequently, even in these affections, than is usually supposed; next to this, an emetic, especially of antimony and ipecacuanha, presents us with one of the most powerful means of effecting this purpose. In either instance this treatment may be followed by a brisk purgative, indeed it frequently happens that a good dose of calomel and colocynth, followed by a common black draught, is sufficient for this purpose. The force of re-action is to be then kept within bounds by smaller doses of the antimonials, perfect rest (especially of the organ affected), determination to the skin, kidneys and bowels; and mercury is to be cautiously administered for the purposes above explained; if the disease shows a disposition to subside steadily under this treatment, the mercury should by no means be unnecessarily pushed to salivation." p. 31. We should not like to place ourselves under Mr. J.'s care, at all events as far as this work gives evidence of his mode of treatment. We are fully convinced that acute inflammations of these tissues—that is inflammations proceeding with rapidity and severity—require most active measures especially directed to the condition of the suffering organ. But treatment, to be successful, must not always be the same for every inflammation proceeding with rapidity and severity; for whilst in some cases general and local bleeding, purging, mercurialization and blisters, &c. are absolutely demanded to check the activity and urgency of the symptoms, in others, tonics, good diet, or even stimulants, are equally required from the very first, in conjunction with local treatment most sedulously and carefully applied. Take, for example, an ordinary attack of acute syphilitic iritis—a disease which is of a decidedly sthenic character, and is most promptly and effectually relieved and cured by the adoption of decided antiphlogistic treatment and mercury, &c., whilst, on the other hand, we may instance that form of acute corneitis which occurs in old persons, or the severe inflammation and ulceration of the cornea which comes on in very depressed states of the system, where upholding the general powers, and subduing the local actions, which tend to the destruction of the organ, by judicious local treatment, hold out the only chance of success.

Our author, however, tells us that, "as respects the antiphlogistic treatment (in syphilitic iritis), it must not be forgotten that this should be pursued with great caution and nicety, as a treatment preservative of the eye, not curative of the disease as a whole. An active antiphlogistic treatment pursued in syphilitic affections, not complicating the eye, would be judiciously condemned as injurious; when an affection of the eye does complicate it, therefore, we should prescribe such treatment with caution." P. 68. Our author is in error because he reasons on false premises. Now we know that antiphlogistic measures—*judiciously applied*—are often of the greatest value in the treatment of many of the stages and symptoms of syphilis, although, of course, not in all; so, in acute syphilitic iritis, the judicious use of antiphlogistic treatment cures the disease without any injury to the constitution—so far from it, that the general state bears, and is benefited by, the means employed. This is the conclusion of general experience, and it would be obviously most absurd to argue against the appropriate use of a remedy by adducing consequences which may arise from its improper or injudicious administration.

We will now turn to the chapter on Cataract, and give our readers an account of the "mixed operation advocated by the author."

"The operation," he tells us, "is no less simple in its description than in its performance. The instrument I use is an ordinary couching-needle with a double cutting edge, as in the operation for solution. The needle being introduced as before described, when speaking of couching and solution, I first endeavour to cut up as much of the lens in situ, without disturbing it from its natural position, as possible. If the cataract be of the softer kind, it of course yields to the needle, and the operation then becomes simply that by solution; if it be so hard that it cannot be cut up, the capsule at least is freely lacerated, and then the point of the needle being raised, is made very gently to press the lens downwards to the extent of a few lines only, and just sufficient to admit of the entrance of a few rays of light. It rarely happens, however, that the cataract is so uniformly hard but that some portion of it at least may be thus cut up. The lens being held in this position for a few seconds, the needle is then carefully withdrawn. Belladonna should now be kept constantly applied to the orbital ridges, for the twofold purpose of freely admitting the access of the aqueous humour to the lacerated lens, and for facilitating the vision of the patient during the process of absorption." 235.

We have often seen an operation somewhat similar, but far superior, to

Mr. Jeaffreson's performed with extraordinary success. It is applicable to a very large class of cases, and is comparatively without risk, although it requires some time for the completion of a cure. The operation is divisible into two portions—the anterior and posterior operation;—in the first, the needle is introduced through the cornea, in front of the iris, (the pupil having been previously dilated,) and the capsule is carefully cut through to a moderate extent, with as much of the structure of the lens itself, as its degree of hardness and other circumstances determine to be proper—taking care not to displace the lens, and never to do too much at one operation. Solution and softening of the lens succeed the admission of the aqueous humour, to an extent proportionate to the less degree of hardness of its structure, and the youth, &c. of the patient; sometimes so much so as not to require a repetition of the operation, at others, demanding two or even three repetitions of the process. When it requires a renewal of these measures it is well to leave about a fortnight between each operation; but, after a longer or a shorter time, the hard nucleus of the lens is entirely removed, it is then that the posterior operation is performed. A needle cutting some distance on its shoulders is introduced behind the iris, and the lens entirely broken up and displaced, leaves the pupil in a short time perfectly clear and unobstructed. Very hard cataracts are better suited for extraction, and very soft or fluid ones may be broken up at once by the posterior operation, but with these exceptions there are no cases which cannot be cured by this method of treatment both safely and pleasantly, if not quickly, for the old proverb is often fulfilled in this disease, that "the more haste the less speed."

Our author's remarks on his mixed operation are also in many particulars applicable to that we have sketched. "The first advantage which it presents is its almost universal applicability; for whilst, in by far the majority of cases, one operation is sufficient to restore the patient to sight, there are indeed but few, if any instances, in which so desirable an end may not be effected by its repetition. Over extraction it presents all those advantages which have been enumerated as belonging to couching, when comparing that with the former operation, but it possesses these advantages, in a degree by so much superior, in that the serious inconvenience sometimes attending the operation for couching, namely, pressure of the lens upon the retina, cannot occur in this mode of proceeding. The operation, moreover, being more gentle in its performance than that of couching, is attended with far less risk of after-inflammation; for whilst in couching, whether by depression or reclination, many of the cells of the vitreous humour are necessarily broken down; in this operation, if carefully performed, a very much less degree of injury is inflicted upon this structure." 250.

The work, we repeat, is unworthy of the author, considering that, according to his own account, the number and extent of his opportunities of acquiring knowledge "have not been surpassed by any oculist however eminent." It clearly shews us that experience is gained not merely by the number of cases a person may see, but by the use he makes of his opportunities; and that our author would probably have written a better work if he had seen but a tithe of the number of cases he boasts of, and paid ten times the attention, he seems to have done, to each case individually.

